# Assessing the Knowledge, Attitudes and Behaviors of Human and Animal Health Students towards Antibiotic Use and Resistance: A Pilot Cross-Sectional Study in the UK

**DOI:** 10.3390/antibiotics7010010

**Published:** 2018-01-30

**Authors:** Oliver James Dyar, Holly Hills, Lara-Turiya Seitz, Alex Perry, Diane Ashiru-Oredope

**Affiliations:** 1 Global Health—Health Systems and Policy, Department of Public Health Sciences, Karolinska Institutet, Tomtebodavägen 18A, 171 77 Stockholm, Sweden; oliver.dyar@ki.se; 2 School of Veterinary Medicine and Science, Sutton Bonington Campus, University of Nottingham, Leicestershire LE12 5RD, UK; svyhh1@nottingham.ac.uk; 3 Imperial College Healthcare NHS Trust, The Bays, South Wharf Road, St Mary’s Hospital, London W2 1NY, UK; lara-turiya.seitz@qehkl.nhs.uk; 4 School of Pharmacy, University College London, 29–39 Brunswick Square, Bloomsbury, London WC1N 1AX, UK; alexandra.perry.13@alumni.ucl.ac.uk; 5 Antimicrobial Resistance Programme, Public Health England, Wellington House, 133–155 Waterloo Road, London SE1 8UG, UK

**Keywords:** antimicrobial, education, training, undergraduate, stewardship, animal health, veterinary, medical, multidisciplinary, one health

## Abstract

The Global Action Plan on Antimicrobial Resistance highlights the importance of training all healthcare professionals. No study has assessed patterns of students’ knowledge, attitudes and practices concerning antibiotic use simultaneously across different healthcare course types. We conducted a cross-sectional multi-center survey among UK students. The survey was advertised through local survey coordinators at 25 universities. The online survey was accessible from 10th October to 17th November 2016 (before European Antibiotic Awareness Day). A total of 255 students from 25 universities participated, including students on medicine, pharmacy, nursing, physician associate, dentistry and veterinary medicine courses. Antibiotic resistance was considered to be a more important global challenge than climate change, obesity or food security (*p* < 0.001). Most students (95%) believed that antibiotic resistance will be a problem for their future practice, but fewer (69%) thought that the antibiotics they will prescribe, administer or dispense will contribute to the problem. A fifth of students felt they had sufficient knowledge of antibiotic use for their future work. Our exploratory study suggests that UK human and animal healthcare students are aware of the importance of antibiotic resistance, but many still have certain misconceptions. Campaigns and improved educational efforts applying behavioral insights methodology could address these.

## 1. Introduction

All human and animal health professions have important roles to play in keeping antibiotics effective [1,2]. It is, therefore, vital that healthcare students are aware of the challenges posed by antibiotic resistance, and that there are investments in training them on topics relevant to responsible antibiotic use in their chosen specialties, as highlighted in the World Health Organization’s Global Action Plan on Antimicrobial Resistance [3]. Although several studies have investigated patterns of knowledge and attitudes on responsible antibiotic use among medical students [4,5] and pharmacy students [6,7], no study so far has attempted to assess these patterns across a broad range of human and animal health courses at the same time using the same survey instrument.

The 2016 Antibiotic Guardian campaign in the United Kingdom aimed to increase awareness of antibiotic resistance among healthcare students, and was launched as part of the national plans for World Antibiotic Awareness Week and European Antibiotic Awareness Day (EAAD). As part of the campaign, but prior to EAAD (18th November), students on selected healthcare courses in the UK were invited to participate in an exploratory online questionnaire on antibiotic use and resistance. The aim was to evaluate these students’ knowledge, attitudes and practices related to antibiotic use and resistance.

## 2. Results

### 2.1. Participants

A total of 255 students from 25 universities in the UK participated. The course types represented were pharmacy (156 students), veterinary medicine (71), medicine (12), dentistry (11), physician associate (3), and nursing (2). Responses for all of these students were included for questions on (i) the perceived importance of antibiotic resistance; and (ii) personal use of antibiotics. A subset of responses was further analyzed which was limited to only responses from students at individual university courses from which ≥10 responses had been obtained. This subset included 210 students from six courses at different universities: three pharmacy courses (134 students included in subset analyses), two veterinary medicine courses (65), and one dentistry course (11). The number of responses for each question varied slightly, as not all students responded to all questions.

### 2.2. Personal Use of Antibiotics

Over a third of students (86/242) had taken oral antibiotics in the previous 12 months. Of these, three had acquired the antibiotics from friends or family, one from an online source, and one leftover from a previous supply.

### 2.3. Knowledge and Perceptions about Antibiotics and Antibiotic Resistance

Most students knew that antibiotics kill both commensal and pathogenic bacteria (88%), and that overuse of antibiotics makes them less effective (96%). Very few (1%) thought that antibiotics killed viruses. Most students (92%) agreed that most coughs, colds and sore throats get better on their own without the need for antibiotics, but 25% of dentistry students still thought that antibiotics were effective against colds. All students were aware that bacteria can become resistant to antibiotics, but many also believed that humans or animals can become resistant (Table 1).

Students considered antibiotic resistance to be a more important global challenge (mean of 9.0, on a scale of 1–10), than climate change (8.4), obesity (8.0), food security (7.7) and gender inequality (7.3), with all comparisons *p* < 0.001.

Students across all healthcare courses considered several factors to be important contributors to antibiotic resistance, including: Too many antibiotic prescriptions (100%); Excessive use of antibiotics in livestock and food production (98%); Poor infection prevention and control practices (96%); Too low dosing of antibiotic prescriptions (83%); Too long durations of antibiotic therapy (75%); Paying too much attention to pharmaceutical advertising (47%).

### 2.4. Actions to Address Antibiotic Resistance

Fewer than half of all students (44%) had heard of either “antimicrobial stewardship” or “antibiotic stewardship”. Students in their third or later year of study were more likely to have encountered the term than those earlier in their courses (61% vs. 18%, *p* < 0.01). Two-thirds of veterinary students had previously heard of the British Veterinary Association’s (BVA) 7-point plan for responsible use of antimicrobials in veterinary practice.

Almost all students (95%) felt that prescribing, dispensing or administering inappropriate or unnecessary antibiotics is professionally unethical. Although most students (95%) believed that antibiotic resistance will be a problem for their future individual practice, fewer (69%) thought that the antibiotics they will prescribe, administer or dispense will contribute to the problem of antibiotic resistance.

### 2.5. Need for More Education

Only a fifth of students felt they had sufficient knowledge of antibiotic use for their future clinical practices. Students in their third or later year of study were more likely to report having sufficient knowledge (28% vs. 11%, *p* < 0.05). Table 2 shows selected topics on which students reported wanting more information.

## 3. Discussion

We conducted a pilot cross-sectional multi-center online survey as part of activities for World Antibiotic Awareness Week (WAAW) and EAAD among students from a range of human and animal health courses in the UK, in order to evaluate students’ knowledge, attitudes and practices related to antibiotic use and resistance. To the best of our knowledge, this is the first study to simultaneously survey students on medical, dentistry, nursing, pharmacy and veterinary medicine courses. Our study is exploratory in nature, and our comparisons are limited by the number of participating students. Many of our results are similar to previous studies conducted among students on medical and pharmacy programs, for example that the majority of students want more education on antibiotic use [5,8]. In this discussion section, we will highlight some of the more novel findings from our study, and those that are particularly relevant for awareness campaigns and educational efforts within healthcare courses.

### 3.1. The Importance of Antibiotic Resistance

Students across different healthcare courses in our survey consistently reported that they believe antibiotic resistance to be a more important global challenge than other key challenges, including climate change, obesity and food insecurity. We placed this question at the beginning of the questionnaire in order to minimize bias based on later questions, although participating students would have been aware that the topic of the survey was antibiotic resistance. We believe it is likely that students’ prioritization of antibiotic resistance was influenced by recent national and international efforts to raise awareness on antibiotic resistance, however no historical studies exist which would have allowed us to make direct comparisons. At a global level, antimicrobial resistance was the theme for World Health Day in 2011, with the World Health Organization considering antibiotic resistance to be among the top three threats to human health [9]. Since then, there have been many high-level efforts in the UK to tackle antibiotic resistance, including public awareness campaigns by Public Health England [10,11,12], and work targeted more towards healthcare professionals. Several of these interventions are highlighted in a number of key documents such as reports by the Chief Medical Officer [13], the UK Antimicrobial Resistance Strategy [14] and the widely reported Review on Antimicrobial Resistance [15]. It is promising if these efforts have translated into widespread awareness of the importance of antibiotic resistance among students across courses in both human and animal health.

### 3.2. What Can Become Resistant to Antibiotics?

In line with high awareness of antibiotic resistance, almost all participating students recognized that overuse of antibiotics makes them less effective, and that bacteria can become resistant to antibiotics. Interestingly, just under a half of participating students in dentistry, pharmacy and veterinary medicine courses believed that humans and animals can also become resistant to antibiotics. Previous studies have found similar potential misconceptions among the general population [16]; students in their early pre-clinical years may have similar baseline beliefs to the general population, and we did find a small reduction in these beliefs among students who were at the later stages of their courses. We are unsure if these are beliefs that antibiotics have direct effects on the cells of humans and animals, which make these cells become resistant; alternatively, students may be expressing the concept that humans or animals can appear resistant to being treated with certain antibiotics by harboring bacteria that are resistant to these antibiotics (e.g., commensal bacteria that have the potential to become pathogenic). Given the crucial role that healthcare professionals play as daily communicators with the general public, we think it is important that students completing their healthcare course are aware of and able to communicate certain basic concepts, for example that humans and animals do not themselves become resistant to antibiotics.

### 3.3. Awareness of Antibiotic Stewardship

As expected, students who were at later stages in their courses were more likely to have heard of antibiotic stewardship. However, many concepts in antibiotic stewardship can be introduced during pre-clinical components of courses, and repeated exposure may reinforce learning [17]. Encouragingly, two-thirds of veterinary students were aware of the BVA 7-point plan, despite this being a relatively new scheme which is not necessarily signposted or taught to those in lower years. A previous study [18] highlighted that although education on antibiotic stewardship is provided to human healthcare and veterinary students across the UK, standardization of this teaching is recommended, which is supported by the results of our survey. Furthermore, the median numbers of hours dedicated to antibiotic stewardship teaching in healthcare courses in the UK is low (17.75 h for medical school courses, 15.5 h for veterinary medicine courses, 12 h for pharmacy courses, 10 h for nursing courses, and 8.5 h for dentistry courses) [18].

This pilot suggests that there is a need for curricula to be developed that comprehensively address the core principles of antimicrobial stewardship. Since all human and animal healthcare professionals have different and complementary roles to play within antimicrobial stewardship, this is an excellent opportunity for interprofessional learning. MacDougall et al. recently developed such a curriculum for pre-clinical medical and pharmacy students, which combined an online learning module with a workshop session, and was supported by faculty from both professions [19]. Students who completed the curriculum had improved knowledge and attitudes about antibiotic resistance; perhaps more importantly, they also had improved attitudes towards interprofessional learning and collaboration.

### 3.4. Further Education

Most students, even in the later years of their courses, still believed they needed more education on antibiotic use and resistance for their future clinical practices. Most dentistry and pharmacy students wanted more information on antibiotic resistance (80% and 70%, respectively), however they were less desiring than veterinary medicine students of information on links between the health of humans, animals and the environment (69% vs. 10% and 42%, respectively), even though it is increasingly apparent that One Health approaches are essential to tackling antibiotic resistance [20,21]. Human healthcare students may lack knowledge and understanding of the role of animal health on human health, or simply perceive this as less practically relevant to the majority of their professional work.

### 3.5. Limitations

This is a predominantly exploratory study of future health professionals undertaking a range of healthcare courses at 25 universities in the UK. A key limitation is that only a small proportion of students at a small group of universities participated, and consequently the majority of the analyses needed to be restricted to students on six different courses. Although we do not have data available on the number of eligible students at participating institutions, we believe that differences in participation are likely due to how the survey was advertised on different courses. For instance, lower participation might be expected if the invitation was sent as part of a newsletter email with many other items, than if the invitation was sent as an email with no other content. Despite these limitations, we have been able to identify some key knowledge gaps among the students participating in our study. Furthermore, our pilot methodology, including the survey instrument developed, should be useful for informing future work. Such studies may aim to make between-course comparisons and to produce more generalizable results, and would need to target a greater number of participating institutions, and a higher number of students within each institution. This may be supported by keeping surveys open for a longer period of time and sending regular invitation reminders.

Although the survey was conducted as part of national plans for WAAW and EAAD, it is unlikely that this will have sensitized students to the topic, since the invitations were not sent in conjunction with any awareness raising materials, and almost all students participated before EAAD (six students (2%) responded after WAAW starting on 14th November but before EAAD on 18th November). Organizing the survey in close connection with a national campaign has several potential advantages: first, networks can be developed to help with both dissemination of a survey and with distribution of campaign materials; second, repeated data collection after a campaign through the same network can allow before and after comparisons to be made to assess the impact of the campaign.

## 4. Materials and Methods

### 4.1. Study Design and Participants

We conducted a cross-sectional multi-center web-based survey among students from a range of healthcare courses in the UK. Local survey coordinators were identified at 25 universities, typically a student from a human or animal health course (a full list of the participating universities is available in the Supplementary Materials Document S1). These coordinators advertised the survey by emailing invitations to students on different human and animal health courses at their university. The survey was accessible on a dedicated Public Health England website between 10th October and 17th November 2016, and participation was voluntary and without compensation.

### 4.2. Questionnaire Development

The survey instrument was developed by a multidisciplinary group of health students and experts on responsible antimicrobial use, informed by the results from previous studies among students from a range of healthcare courses. The survey consisted of 25 questions, including sections on demographics; knowledge, attitudes and practices towards antibiotic use and resistance; and awareness of antimicrobial stewardship. The survey was pilot tested by participants in the 2016 Antibiotic Guardian student-planning group. The survey instrument is included in the Supplementary Materials Document S2.

### 4.3. Statistical Analyses

We excluded all individual responses in which participants had completed fewer than half of the questions. With the exception of questions on the perceived importance of antibiotic resistance and personal use of antibiotics, only a subset of responses was analyzed, limiting participation to individual university courses from which ≥10 responses had been obtained. Descriptive and inferential statistics (*t*-tests) were used to analyze the data, with all analyses conducted in Microsoft Excel 2016 (Microsoft Inc., Redmond, WA, USA). Where appropriate, results are presented by healthcare course type, and by the stage of students within their respective courses (first and second year vs. third year or higher).

### 4.4. Ethical Approval

Ethical approval was not considered necessary for this study. The voluntary survey was part of evaluating activities for World Antibiotic Awareness Week and EAAD in the UK.

## 5. Conclusions

Our exploratory study suggests that students across a broad range of healthcare courses in the UK are aware of antibiotic resistance, and believe it is an extremely important global challenge. We identified some misunderstandings, and that most students feel a need for more education on antibiotic use and resistance. Campaigns such as Word Antibiotic Awareness Week and European Antibiotic Awareness Day, as well as the Antibiotic Guardian campaign, are raising the profile of this topic among students and healthcare professionals, and play a vital role in the effort to provide this extra education. Furthermore, within universities interprofessional learning approaches are an innovative means to improving both topic-specific knowledge as well as interdisciplinary teamwork skills.

## Figures and Tables

**Table 1:** Healthcare students’ awareness of what can become resistant to antibiotics.

Agree with Statement (%)						
Statement	All Students (*n* = 165)	Pharmacy Students (*n* = 104)	Veterinary Medicine Students (*n* = 53)	Dentistry Students (*n* = 8)	<3rd Year Students (*n* = 68)	≥3rd Year Students (*n* = 97)
Bacteria can become resistant to antibiotics	100%	100%	100%	100%	100%	100%
Humans can become resistant to antibiotics	41%	48%	28%	25%	46%	37%
Animals can become resistant to antibiotics	44%	50%	34%	25%	51%	39%

**Table 2:** Topics on which healthcare students want more information.

Want More Information (%)				
Topic	All Students (*n* = 204)	Pharmacy Students (*n* = 132)	Veterinary Medicine Students (*n* = 62)	Dentistry Students (*n* = 10)
How to use antibiotics	25%	33%	11%	20%
Resistance to antibiotics	63%	70%	47%	80%
Prescription of antibiotics	45%	54%	27%	30%
Links between the health of humans, animals and the environment	49%	42%	69%	10%

## Supplementary Materials

The following are available online at http://www.mdpi.com/2079-6382/7/1/10/s1, Document S1: List of all participating universities; Document S2: Survey instrument.
