# Supplementary material for: Assessing the Knowledge, Attitudes and Behaviors of Human and Animal Health Students towards Antibiotic Use and Resistance: A Pilot Cross-Sectional Study in the UK
Source: Antibiotics (Basel). 2018 Jan 30;7(1):10. doi: 10.3390/antibiotics7010010 (PMC5872121; doi:10.3390/antibiotics7010010)
Supplement: Supplementary file 1 [file antibiotics-07-00010-s001.zip › Document S2.pdf]

Public Health England

Evaluation of WAAW and Antibiotic Guardian campaign for Healthcare Students

Healthcare Students' perceptions of antibiotic resistance

This survey for undergraduate healthcare students (medicine, pharmacy, nursing and veterinary medicine/nursing) is part of our evaluation of activities for World Antibiotic Awareness Week (WAAW) 2016. Participation is completely voluntary.

The survey should take five to ten minutes to complete. All responses are anonymous and will be stored confidentially.

Please complete before 31 October 2016.

Thank you

Dr Diane Ashiru-Oredope
Chair, Antibiotic Guardian Campaign and WAAW

1. At which University do you study?
   - Kings College London
   - UCL
   - University of Manchester
   - University of Nottingham
   - Durham University
   - Royal Veterinary College
   - Liverpool John Moores
   - Other, please specify

2. Which degree course are you studying?
   - Dentistry
   - Medicine
   - Nursing
   - Pharmacy
   - Veterinary Medicine
   - Veterinary Nursing
   - Other, please specify

3. Which year of study are you in?
   -- None --

4. What gender do you most identify with?
   -- None --

5. Please indicate your age:
   - 12-17 years old
   - 18-24 years old
   - 25-34 years old
   - 35-44 years old
   - 45-54 years old
   - 55-64 years old
   - 65 years or older
   - prefer not to say

6. Do you have any preferred place to work in the future?
   - Secondary care (e.g. hospital)
   - Primary care (e.g. community settings, general practice)
   - Industry
   - Academia
   - Not sure
   - Other, please specify

7. What is your email address? (We will share a summary of the results and love to get additional feedback after WAAW)

Public Health England

Evaluation of WAAW and Antibiotic Guardian campaign for Healthcare Students

Personal use of antibiotics

8. In the last 12 months, have you taken any antibiotics orally such as tablets, powder or syrup?
   -- None --

9. How did you obtain the last course of antibiotics that you used? (select one)
   -- None --

10. On which topics, if any, would you like to receive more information?
    - How to use antibiotics
    - Resistance to antibiotics
    - Medical conditions for which antibiotics are used
    - Prescription of antibiotics
    - Links between the health of humans, animals and the environment
    - Other, please specify

Public Health England

Evaluation of WAAW and Antibiotic Guardian campaign for Healthcare Students

Knowledge

11. How important do you think the following challenges are in the world today? (scale of 1 [not at all important] to 10 [extremely important] for each)

	1	2	3	4	5	6	7	8	9	10
Climate change										
Food security										
Antibiotic resistance										
Gender inequality										
Obesity										

12. Are the following statements true or false? (Options = True/False/I don't know)

	True	False	I don't know
Antibiotics kill both good and bad bacteria	○	○	○
Antibiotics kill viruses	○	○	○
Antibiotics are effective against colds	○	○	○
Overuse of antibiotics makes them become ineffective	○	○	○
Taking antibiotics often has side-effects such as diarrhoea	○	○	○
Bacteria can become resistant to antibiotics	○	○	○
Humans can become resistant to antibiotics	○	○	○
Animals can become resistant to antibiotics	○	○	○
A pig that has been treated with	○	○	○

antibiotics can be slaughtered for meat purposes the next day [Vet students only]

13. Where do you think most antibiotics in human healthcare are used?

-- None --

14. Please state whether you think each of the following drugs are antibiotics, anti-inflammatories, or other types of drug:

	Antibiotic	Anti-inflammatory	Other
Amoxicillin	○	○	○
Simvastatin	○	○	○
Thyroxine	○	○	○
Cephradine	○	○	○
Aspirin	○	○	○
Erythromycin	○	○	○
Ibuprofen	○	○	○

15. How many people die because of infections from resistant bacteria in Europe each year?

-- None --

16. How many human deaths are predicted to happen globally in 2050 due to antimicrobial resistance, if current trends continue?

-- None --

17. What is the difference between an antimicrobial and an antibiotic? (select one)

- ○ Antibiotics refers to drugs that kill bacteria, whereas antimicrobials includes drugs that kill viruses, fungi or bacteria
- ○ Antibiotics refers only to naturally occurring compounds, it does not include synthetic compounds
- ○ There is no difference between an antibiotic and an antimicrobial
- ○ Don't know

18. Have you ever heard of the term 'antibiotic stewardship' or 'antimicrobial stewardship'?

-- None --

19. Have you heard of the British Veterinary Association (BVA) 7 point plan for responsible use of antimicrobials in veterinary practice?

-- None --

20. Which of the following is NOT effective in preventing the emergence or spread of antimicrobial resistant organisms? (select one)

- ☐ Good hand hygiene
- ☐ Treating infections for a longer period to prevent resistance

- ☐ Isolation of patients colonised with resistant organisms such as MRSA
- ☐ Avoiding the use of antibiotics for treatment of colds and flu
- ☐ Take up the offer of the flu vaccine

21. How can antimicrobial-resistant bacteria spread to humans? (select one or all that apply)

- ☐ Contact with a person who has an antibiotic-resistant infection
- ☐ Contact with something that has touched a person who has an antibiotic resistant infection (e.g. a health-workers' hands or instruments in a health facility with poor hygiene
- ☐ Contact with a live animal, food or water carrying antibiotic-resistant bacteria

22. What is the best way to clean your hands to stop the spread of bacteria? (select one)

- ○ Rinsing with hot water
- ○ Use a hand sanitising gel/foam
- ○ Wash thoroughly with soap and water
- ○ Wearing gloves means I don’t need to

Public Health England

Evaluation of WAAW and Antibiotic Guardian campaign for Healthcare Students

Attitudes

23. Which of the following potential contributors are important to antibiotic resistance (for each one: very important / important / not important at all / unsure)?

	Very important	Important	Not important at all	Not sure
Too many antibiotic prescriptions	○	○	○	○
Too many broad spectrum antibiotics used	○	○	○	○
Too long durations of antibiotic treatment	○	○	○	○
Dosing of antibiotics too low	○	○	○	○
Excessive use of antibiotics in livestock and food production	○	○	○	○
Poor infection prevention and control practices	○	○	○	○
Paying too much attention to pharmaceutical representatives/advertising	○	○	○	○
Public awareness of	○	○	○	○

antibiotic resistance

24. The following are statements some people have made regarding antibiotics. To what extent you agree or disagree with the following?

	Strongly disagree	Disagree	Neither agree/nor disagree	Agree	Strongly agree
Bacteria that are resistant to antibiotics spread easily from person to person	○	○	○	○	○
Most coughs, colds and sore throats get better on their own without the need for antibiotics	○	○	○	○	○
Healthy people can carry antibiotic-resistant bacteria	○	○	○	○	○
Biosecurity is important in food production	○	○	○	○	○
When there is poor biosecurity, prophylactic antibiotics are an appropriate cover	○	○	○	○	○

25. Please answer each of the following questions:

	Yes	No	Unsure
Do you believe that prescribing, dispensing or administering inappropriate or unnecessary antibiotics is professionally unethical?	○	○	○
Do you think we have enough antibiotics under development at the moment to keep up with the problem of resistance?	○	○	○
Do you think that antibiotic resistance is a national problem?	○	○	○
Do you think that antibiotic resistance will be a problem for your future individual practice?	○	○	○
Do you think that antibiotic resistance will be a greater	○	○	○

problem later in your career than it is today?			
Do you believe that the antibiotics you will prescribe/administer/dispense will contribute to the problem of antibiotic resistance?	○	○	○
Do you feel you have sufficient knowledge on antibiotic use for your future practice?	○	○	○

26. How important do you think resistance is as a problem among the following microbes: (scale 1 [not at all important] to 10 [extremely important])

	1	2	3	4	5	6	7	8	9	10
Bacteria (excluding tuberculosis)										
Tuberculosis										
Fungi										
Viruses										

Public Health England

Evaluation of WAAW and Antibiotic Guardian campaign for Healthcare Students

27. Thank you very much for completing survey. Please use this section to provide additional comments or questions. Please click DONE once complete
