# Supplementary material for: Assessing the Knowledge, Attitudes and Behaviors of Human and Animal Health Students towards Antibiotic Use and Resistance: A Pilot Cross-Sectional Study in the UK
Source: Antibiotics (Basel). 2018 Jan 30;7(1):10. doi: 10.3390/antibiotics7010010 (PMC5872121; doi:10.3390/antibiotics7010010)
Supplement: Supplementary file 1 [file antibiotics-07-00010-s001.zip › Document S1.docx]

**Participating universities**

***Assessing the knowledge, attitudes and behaviours of human and animal health students towards antibiotic use and resistance: a pilot cross-sectional study in the UK***

Aston University

Durham University

Edinburgh University

King’s College London

Liverpool John Moores University

Medway School of Pharmacy, University of Greenwich

Newcastle University

Royal Veterinary College

University College of London

University of Bath

University of Bradford

University of Brighton

University of Bristol

University of Cambridge

University of Central Lancashire

University of East Anglia

University of Huddersfield

University of Leeds

University of Liverpool

University of Manchester

University of Nottingham

University of Portsmouth

University of Reading

University of Warwick

University of West London
